# The Potential Cost-Effectiveness of Pre-Exposure Prophylaxis Combined with HIV Vaccines in the United States

**DOI:** 10.3390/vaccines5020013

**Published:** 2017-05-24

**Authors:** Blythe J. S. Adamson, Josh J. Carlson, James G. Kublin, Louis P. Garrison

**Affiliations:** 1 Pharmaceutical Outcomes Research and Policy, Department of Pharmacy, University of Washington, Seattle, WA 98115, USA; carlsojj@uw.edu (J.J.C.); lgarrisn@uw.edu (L.P.G.J.); 2 HIV Vaccine Trials Network, Fred Hutchinson Cancer Research Center, Seattle, WA 98109, USA; jkublin@fredhutch.org; 3 Department of Global Health, University of Washington, Seattle, WA 98115, USA

**Keywords:** economic evaluation, mathematical modeling, HIV vaccines, pre-exposure prophylaxis, cost-effectiveness

## Abstract

This economic evaluation aims to support policy-making on the combined use of pre-exposure prophylaxis (PrEP) with HIV vaccines in development by evaluating the potential cost-effectiveness of implementation that would support the design of clinical trials for the assessment of combined product safety and efficacy. The target study population is a cohort of men who have sex with men (MSM) in the United States. Policy strategies considered include standard HIV prevention, daily oral PrEP, HIV vaccine, and their combination. We constructed a Markov model based on clinical trial data and the published literature. We used a payer perspective, monthly cycle length, a lifetime horizon, and a 3% discount rate. We assumed a price of $500 per HIV vaccine series in the base case. HIV vaccines dominated standard care and PrEP. At current prices, PrEP was not cost-effective alone or in combination. A combination strategy had the greatest health benefit but was not cost-effective (ICER = $463,448/QALY) as compared to vaccination alone. Sensitivity analyses suggest a combination may be valuable for higher-risk men with good adherence. Vaccine durability and PrEP drug prices were key drivers of cost-effectiveness. The results suggest that boosting potential may be key to HIV vaccine value.

## 1. Introduction

HIV treatment and prevention in the United States (USA) requires substantial societal resources and the treatment of HIV-infected patients is generally cost-effective. Based on economic models, if treated, a person infected with HIV at age 35 in the U.S. will, on average, suffer from lower quality and length of life and accumulate $247,500 (2015 USD) more in lifetime medical costs compared to people who are not HIV infected [1,2,3]. Federal funds in 2016 allocated $20 billion for domestic HIV care and $1 billion for domestic HIV prevention [4]. To date, only one drug has a Food and Drug Administration (FDA)-approved indication for prevention. Truvada^®^ is a single-pill fixed-dose antiretroviral combination of tenofovir disoproxil fumarate (TDF) and emtricitabine (FTC) launched in 2004 to treat HIV (Gilead Sciences, Inc., Foster City, CA, USA). The FDA approval expanded Truvada’s^®^ indication in 2012 as a safe and effective daily oral medication to reduce the risk of sexually acquired HIV infection, a form of pre-exposure prophylaxis (PrEP). PrEP studies including Pre-exposure Prophylaxis Initiative (iPrEX), Pre-exposure prophylaxis to prevent the acquisition of HIV-1 infection (PROUD), France Recherche Nord et Sud Sida- HIV et Hépatites (ANRS), Intervention Préventive de l’Exposition aux Risques avec et pour les Gays (IPERGAY), and Kaiser Permanente studies reported efficacy ranging from 42% to 99% with adherence strongly correlated with effectiveness [5,6,7,8,9]. Side effects in some patients include diarrhea, nausea, liver toxicity, and bone mineral density loss. By 2015, Truvada^®^ had the largest market share (17%) of all HIV drugs with no competing HIV drugs on the market for prophylaxis. The potential market for PrEP is estimated as 1.2 million people, including 25% of the estimated 4.5 million men who have sex with men (MSM) in the U.S. [10,11,12]. The average wholesale price of Truvada^®^ was $1646 for a 30-day supply in 2015 whether used for prevention or treatment of HIV [13].

HIV vaccines in development, and currently in clinical trials, may eventually be used in place of or in combination with PrEP. A Phase III study in Thailand with more than 16,000 participants (labeled as RV144 and referred to as “the Thai trial” in this paper) established an HIV vaccine candidate with an average 31% preventive efficacy over three years [14]. Immunogenicity results from a follow-up study of RV144 participants re-vaccinated years later suggests boosting may be effective [15]. A National Institute of Allergy and Infectious Diseases (NIAID)-funded confirmatory trial (HVTN 702) in South Africa was launched in 2017 to evaluate the safety and preventive efficacy of ALVAC-HIV (vCP2438) vaccine prime with bivalent subtype C gp120/MF59 boosts (see descriptions in Appendix Table A1) [16,17]. Compared to the Thai trial, the HVTN 702 vaccine regimen, which matches the HIV sub-type circulating in Southern Africa, replaces alum with the potentially more potent adjuvant MF59 and adds a fifth dose at 12 months to the regimen schedule [17]. This pivotal HIV vaccine trial hypothesizes an average vaccine efficacy (VE) of 50% over 24 months, and is scheduled to be completed in 2021.

Previous economic evaluations have separately examined the cost-effectiveness of PrEP or HIV vaccines in the USA but none have modeled the potential outcomes when combining these products [18,19,20,21,22,23], as shown in a recent review of HIV vaccine cost-effectiveness studies [24]. For the treatment of HIV, Truvada^®^ is highly cost-effective when used in combination with other drugs, but the cost-effectiveness estimates for prevention are mixed in reviews [21,25,26]. If an HIV vaccine is launched in the USA, experts may consider modifying PrEP clinical guidelines to inform the most efficient use in combination with HIV vaccines [27]. This analysis is the first to assess the potential cost-effectiveness of combining PrEP with an HIV vaccine in comparison to either alone for MSM in the U.S. Specifically, the objective of our study is to identify the potential cost-effectiveness of HIV vaccines co-administered with PrEP and to investigate thresholds for vaccine characteristics for efficient use in MSM. The findings have implications not only for potential uptake but also for the prioritization of PrEP and vaccine candidates progressing through the clinical development pipeline.

## 2. Materials and Methods

This modeling study followed methodology recommendations from the Second Panel on Cost-Effectiveness in Health and Medicine and meets standardized reporting requirements from the Consolidated Health Economics Evaluation Reporting Standards (CHEERS) statement [28,29].

### 2.1. Study Population

The analysis evaluated policy strategies for potential implementation of HIV prevention interventions in a cohort of HIV-negative MSM in the USA The base-case analysis models men of average age 30 years until death, i.e., a lifetime horizon. A sub-group analysis focuses on a cohort of “high-risk” men, defined as having anal sex without a condom in the last 12 months, based on clinical practice guidelines recommending PrEP [27].

### 2.2. Model Overview

**Figure 1:**
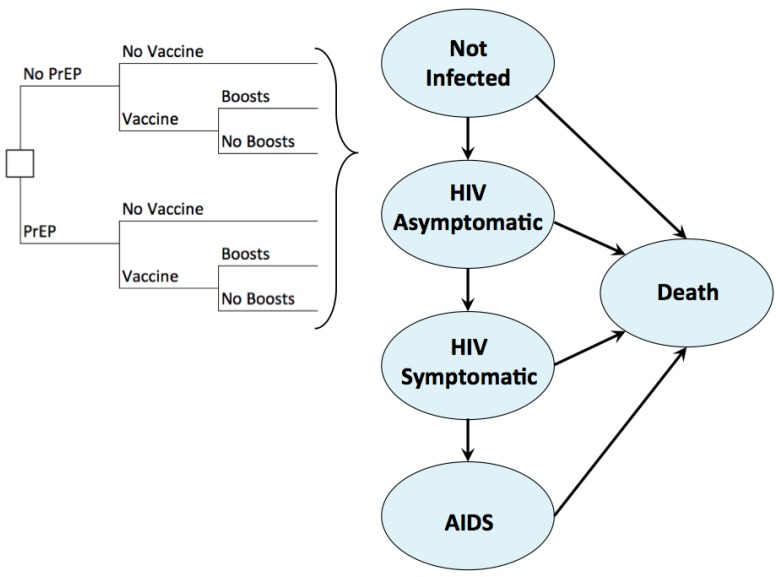
Simplified conceptual diagram of health states in the Markov model.

We developed a Markov health-state transition model of HIV infection and disease progression and used the model to estimate clinical benefits, total costs, and the cost-effectiveness of strategies delivering HIV vaccines and PrEP alone or in combination. We developed a model based on previous works by Sanders et al. and Bayoumi et al. [30,31]. Importantly, we add functions to describe PrEP of varying duration and HIV vaccines with waning efficacy and boosting. Health states, seen in Figure 1, are connected by difference equations solved at monthly time steps. Parameter values were informed by the most recent peer-reviewed literature. The HIV prevention strategies evaluated include: PrEP alone, HIV vaccines alone, co-administration of PrEP and HIV vaccines, and a reference base-case of standard HIV prevention without PrEP or vaccines. An Impact Inventory (Appendix Table A2) catalogues the intervention costs and effects within and outside the healthcare sector and identifies components included in this analysis [28].

### 2.3. Model Inputs

Table 1 summarizes the key model inputs.

#### 2.3.1. HIV Incidence

**Figure A1:**
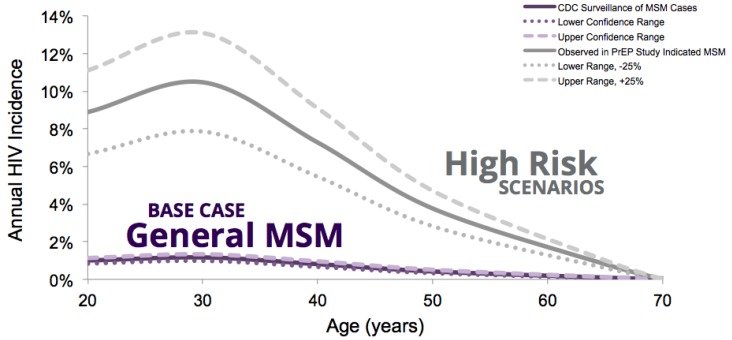
Incident HIV infections among men-who-have-sex-with-men (MSM) in the United States, based on 2012 CDC surveillance data [32].

HIV-negative men entering the model had an age-dependent risk of infection. The input values for HIV incidence were calculated from Centers for Disease Control (CDC) surveillance data on newly detected cases and population sizes from the U.S. Census Bureau (Table 1) [32,33]. Cross-sectional MSM incidence was extrapolated to future years. Given the uncertainty in HIV-incidence among PrEP-indicated MSM, we scaled the observed trend by age to match the incidence levels observed in the PROUD study participants to represent the high-risk sub-group, also with HIV incidence dependent on age (Figure A1) [8]. For example, at the age of greatest average risk, 30–34 years, the HIV incidence input value for general MSM was 1.2 infections per 100 person-years and for high-risk MSM was 10.5 infections per 100 person-years. Incidence rates were converted into the probability of infection in a monthly time step. 

#### 2.3.2. Clinical Inputs

Newly infected HIV patients progressed over time through health states defined by CD4+ T-cell count categories (>500, 200–499, and <200 T-cells per mL). The probability of monthly transitions through progressing health states represent population averages based on the published literature (Table 1). Age-and gender-specific baseline mortality rates were calculated from 2010 United States Life Tables [34]. Based on the Strategies for Management of Antiretroviral Therapy (SMART) Study and Evaluation of Subcutaneous Proleukin in a Randomized International Trial (ESPRIT) in well-controlled HIV infected individuals, patients with CD4 counts ranging from 200–500 had a 1.77 times increased hazard of non-AIDS death (95% CI: 1.17–2.55)compared to the general population, but those with CD4 >500 had no increased risk of death [35]. Patients with CD4 <200 could die from AIDS in addition to their baseline risk of death from other causes [36].

#### 2.3.3. Health State Utility

We identified preference-based utility weights (Table 1) for HIV health states defined by CD4 T-cell count categories (Figure 1) in the published literature [30,37,38,39,40]. Utilities for uninfected men are stratified by age and based on healthy males in the general U.S. population [37]. To account for the range of adverse events associated with PrEP, such as bone mineral density loss, the time using PrEP had a utility decrement of 0.008 applied to each quarter of use (ranging 0–0.1 in the sensitivity analyses). To adjust for potential HIV vaccine reactogenicity, men lost the equivalent of one quality-adjusted day at the time of each vaccine injection.

#### 2.3.4. Intervention Effectiveness

**Figure 2:**
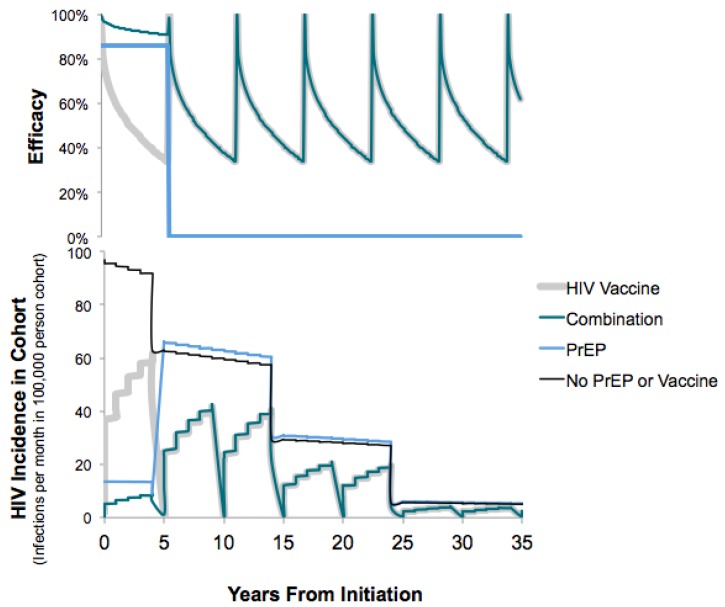
Efficacy and epidemic impact of HIV prevention strategies. The top panel shows the average efficacy over time given each strategy and the lower panel shows the number of new infections per month per 100,000 persons in the cohort of men. Baseline HIV incidence declines with age categories.

**Figure A2:**
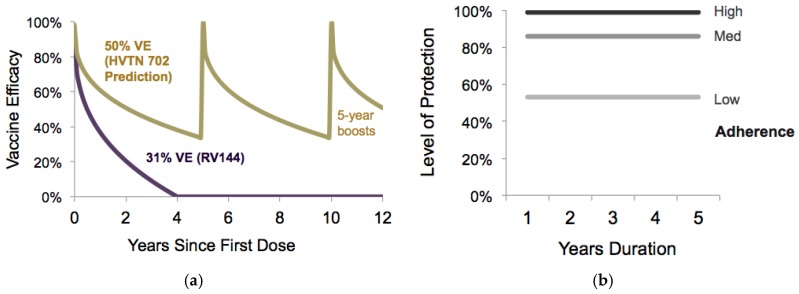
Percent reduction in HIV infection for HIV vaccines and PrEP as modeled in the analysis. Intervention efficacy assumptions for (**a**) HIV vaccine efficacy decaying over time with boosting every 5 years and (**b**) PrEP by the level of adherence. Abbreviations: PrEP, Pre-exposure prophylaxis; VE, vaccine efficacy.

We define the standard of care as routine HIV testing, risk reduction counseling, and no availability of PrEP or HIV vaccines. The base-case PrEP strategy assumes average adherence corresponding to 86% effectiveness in reduction in HIV incidence, based on the Partners in Prevention and IPERGAY studies, and an average duration of 5 years [8,41,42]. Scenarios with lower adherence to PrEP drugs had 53% efficacy, based on iPrEX study findings [6,43], and scenarios with higher PrEP adherence had 99.9% effectiveness based on an observational study of PrEP users in the Kaiser Permanente Health System [9]. Ranges of PrEP duration (0–10 years) and effectiveness (40–99.9%) are explored in the sensitivity analysis. Base-case HIV vaccination resembled the HVTN 702 regimen with a five-dose series administered over 12 months (Figure A2). We modified the proportional hazards model by Hankins et al., originally fitted to the 31% vaccine efficacy (VE) observed in the Thai trial [44], to describe the expected waning over time with an average of 50% VE at 24 months as expected in HVTN 702. The time-dependent reduction in the likelihood of HIV acquisition following a complete HIV vaccine series followed the equation

$$VE_{t}=1-\exp(-2.88+0.76\times \;\left(\log\left(\left(t+0.001\right)\times 30\right)\right)$$

where *t* is the time in months since the first dose of the most recent vaccination series (see Figure 2 and Appendix Figure A2). We assumed that re-vaccination five years later boosted immunity to the initial levels followed by the same rate of exponential decay in protection from infection [45]. The PrEP-Vaccine combination strategy assumes the cohort of MSM initiates PrEP at the time of vaccination, and then they continue PrEP for five years and receive HIV vaccine boosts every 5 years (varying 0–10 years in sensitivity analyses). Figure 2 shows the average efficacy for each strategy over time. We assume that the combined effectiveness is multiplicative,

$$p_{t}=\left(1-RR_{PrEP}\right)\times \left(1-VE_{t}\right)$$

where *p_t_* is the reduction in likelihood of HIV infection from the combination at month *t*.

#### 2.3.5. Costs

HIV-related healthcare costs were projected from a US health care payer perspective over the patient lifetime horizon. Inputs were derived from the published literature and adjusted to 2015 US dollars using the medical consumer price index [46,47]. A cost study of USA healthcare expenditures among HIV patients, stratified by CD4-count categories, provided the distribution and types of healthcare expenditures for HIV patients [48].

PrEP users incurred costs from quarterly clinic visits with an HIV antibody test, other sexually transmitted infections diagnostic tests, and the measurement of blood urea nitrogen and serum creatinine levels. PrEP drugs cost $1646 per month, based on the average wholesale price for a 30-day supply of Truvada^®^ in 2015 [13]. As the launch price for an HIV vaccine is unknown, we benchmarked on the price per dose of other FDA-approved vaccines to prevent other sexually transmitted infections [49] and consulted expert opinion. We assumed an HIV vaccine launch price of $500 per dose, totaling $2500 for the five-dose series. The cost per vaccine dose ranged from $100–$1000 in the sensitivity analysis.

### 2.4. Model Outputs

The hypothetical cohort of men was followed from the time of intervention until death. Patient outcomes are reported as per-person averages, and include lifetime discounted healthcare costs, lifetime probability of HIV infection, expected life years (LYs), and expected quality-adjusted life years (QALYs). To reflect both the length and quality of life, QALYs were calculated as the sum of the monthly survival time multiplied by the utility value for the corresponding health state. Total costs and QALYs are discounted 3% annually to reflect preferences for present as compared to future gains, also known as opportunity cost, following the guidelines from the Second US Panel on Cost-Effectiveness [54,55].

### 2.5. Cost-Effectiveness Analysis

For the primary economic endpoint, we estimated the incremental cost-effectiveness ratio (ICER) for each scenario using the equation

$$\mathrm{ICER}=\;\frac{{\mathrm{Costs}}_{\mathrm{intervention}}\;–{\;\mathrm{Costs}}_{\mathrm{standard}\;\mathrm{care}}}{{\mathrm{QALYs}}_{\mathrm{intervention}}\;-{\;\mathrm{QALYs}}_{\mathrm{standard}\;\mathrm{care}}}.$$

To interpret cost-effectiveness, we defined a cost-effectiveness threshold for the US health care payer with an assumed willingness to pay for health gains. Consistent with recommendations from the Second Panel on Cost-Effectiveness in Health and Medicine and several pharmaceutical value frameworks, we interpret ICERs <$50,000/QALY as highly cost-effective, $50,000–$150,000/QALY as cost-effective, and >$150,000/QALY as unlikely to be cost-effective, given a threshold of 1–3 times the gross domestic product (GDP) per capita in the U.S. [28,56]. If an intervention strategy had a lower ICER and greater total health gains, it ruled out the less cost-effective strategies by “extended dominance” [57]. HIV incidence and HIV vaccine price varied in threshold analyses to identify the maximum value at which the strategy remained cost-effective when all other parameter values remain fixed. As a secondary economic endpoint, the incremental cost per HIV infection averted was estimated for each strategy.

### 2.6. Sensitivity Analysis

One-way (univariate) sensitivity analyses were performed using the upper and lower ranges of each input, holding all other variables constant, to explore the model’s sensitivity to uncertainty in individual parameters (Table 1). We explored more than 500 scenarios to evaluate policy relevant cases of interest to decision-makers. Scenarios projected impacts at varying ages for the initiation of each intervention, lengths of PrEP duration, levels of PrEP adherence, and frequency of vaccine boosting. A sub-group analysis estimated the cost-effectiveness of the interventions for high-risk MSM.

A multivariate probabilistic sensitivity analysis (PSA) evaluated the combined parameter uncertainty in the model. We selected and fitted distributions for each model parameter and followed gamma for costs, beta for utilities, and normal for risk reduction using the method of moments. Monte Carlo simulations generated a unique set of input values based on random draws from these distributions and re-estimation of model outcomes as 1000 simulations per strategy.

## 3. Results

### 3.1. Base Case

#### 3.1.1. Clinical Outcomes

The cohort with standard preventive care (no PrEP or HIV vaccine) had a lifetime HIV risk of 171 cases/1000 MSM (see black line in the lower panel of Figure 2). Delivering PrEP for five years reduced the lifetime risk of HIV by 25% and gained an average 0.38 lifetime QALYs per person (Table 2). HIV vaccines alone (with waning immunity with an average 50% VE over 3 years, boosting every 5 years) reduced the risk of HIV in the cohort to 88 cases/1000 men (48% reduction compared to the standard care) and gained an additional 0.14 lifetime QALYs compared to PrEP alone (see the grey lines in Figure 2). The combination of PrEP with an HIV vaccine achieved the largest health gains and an incremental 0.19 lifetime QALYs per person (see the dark blue lines in Figure 2) compared to vaccination alone.

#### 3.1.2. Costs

**Figure 3:**
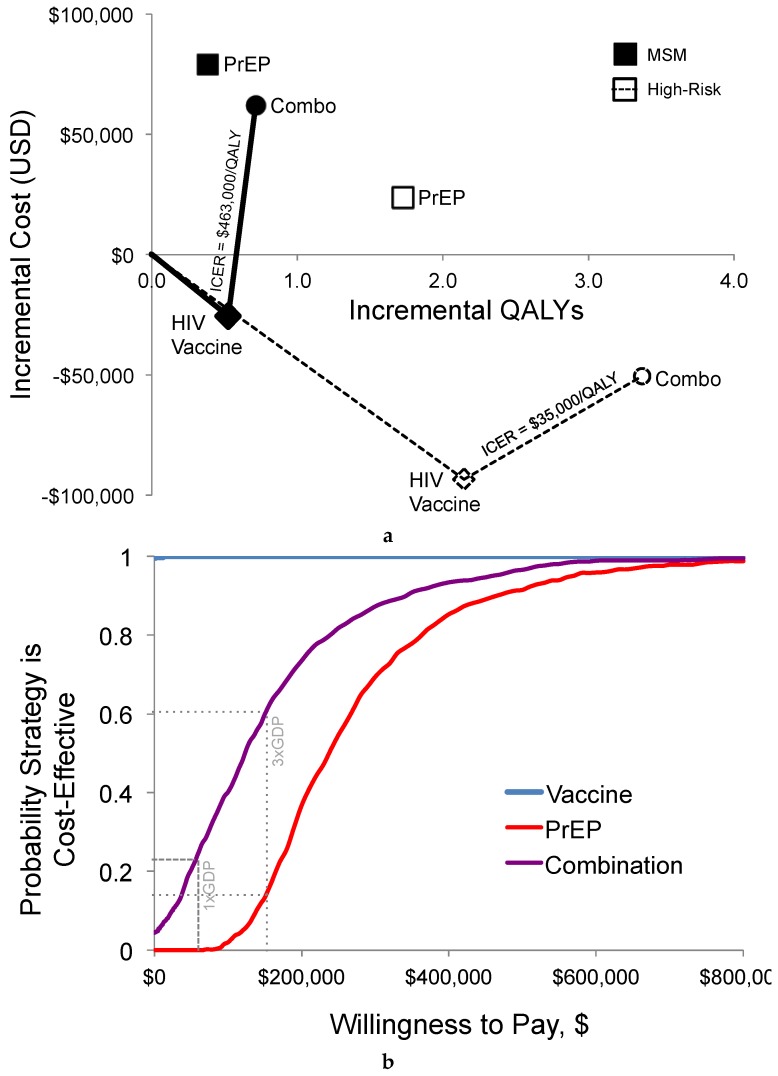
Cost-effectiveness results for potential prevention policies. The cost-effectiveness plane in panel (**a**) shows the origin representing standard preventative care, y-axes as the average incremental lifetime discounted costs per-person, and x-axis of QALYs gained for each policy strategy as compared to standard prevention. A solid line represents the cost-effectiveness frontier for the base-case population of all MSM and a dashed line connects a high-risk scenario. Panel (**b**) shows results from the probabilistic sensitivity analysis as a cost-effectiveness acceptability curve. Abbreviations: QALYs, quality-adjusted life-years; ICER, incremental cost-effectiveness ratio; MSM, men who have sex with men; PrEP, pre-exposure prophylaxis.

HIV prevention and treatment-related healthcare for the cohort using PrEP (average duration of five years with 86% efficacy) cost an average $78,884 more per person than the standard care over the lifetime (Table 2 and Figure 3). The HIV vaccine strategy cost $21,057 less per person than the standard care. The combination of PrEP with vaccination cost $66,558 more than the standard care and $12,326 less than PrEP alone. Over time, as patients aged, the added cost of each re-vaccination had a smaller marginal return in terms of reducing anti-retroviral therapy (ART) drug costs.

#### 3.1.3. Cost-Effectiveness

HIV vaccination alone dominated PrEP, as the vaccine had greater health gains and lower total costs than PrEP (Table 2 and Figure 3). Vaccines dominated standard care by $40,224 per QALY. The combination of PrEP with HIV vaccines had an ICER of $463,448 per QALY gained, as compared to HIV vaccines alone, and would not be cost-effective even given the upper-bound threshold of $150,000 per QALY.

### 3.2. Sensitivity Analysis

**Figure 4:**
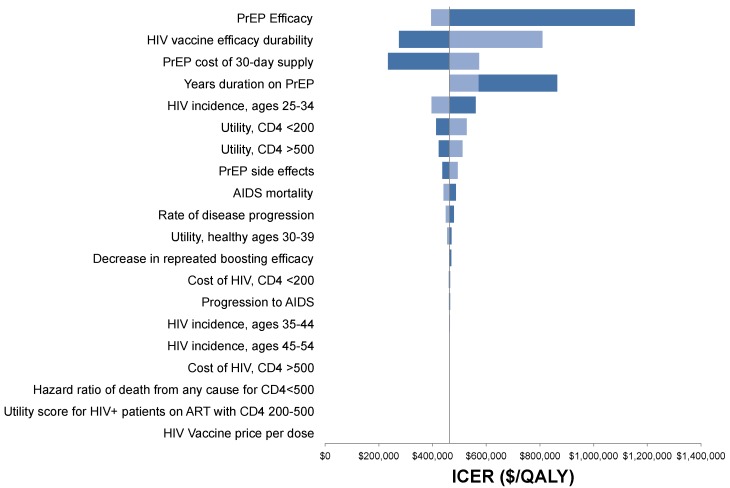
Tornado diagram of the one-way sensitivity analysis showing the impact of minimum and maximum parameter ranges on the ICER of the combination strategy versus HIV vaccines alone. Univariate sensitivity of the PrEP duration shows that 1 year or 10 years on PrEP in the combination strategy have larger ICERs than the base case assumption of 5 years duration, because the balance of lifetime PrEP costs and benefits is closer to the optimization of duration at 5 compared to 1 or 10 years.

Cost-effectiveness findings were most sensitive to HIV incidence rates and PrEP drug costs in the univariate analyses (Figure 4). The cost-effectiveness of the PrEP/vaccine combination was more sensitive to the rate of decay in VE (also known as durability) than to the uncertainty in the cost or duration of PrEP. Using a threshold analysis, we estimate the maximum cost-effective price of PrEP drugs would be no more than $893 per 30-day supply, corresponding to a 50% reduction in the average wholesale price of Truvada^®^ in 2015. At the largest hypothesized range of HIV vaccine price—$5000 per series and per boost—the vaccines resulted in lower lifetime health system costs than the standard prevention.

#### 3.2.1. Scenarios

**Figure 5:**
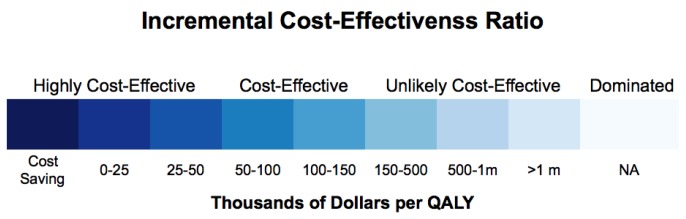

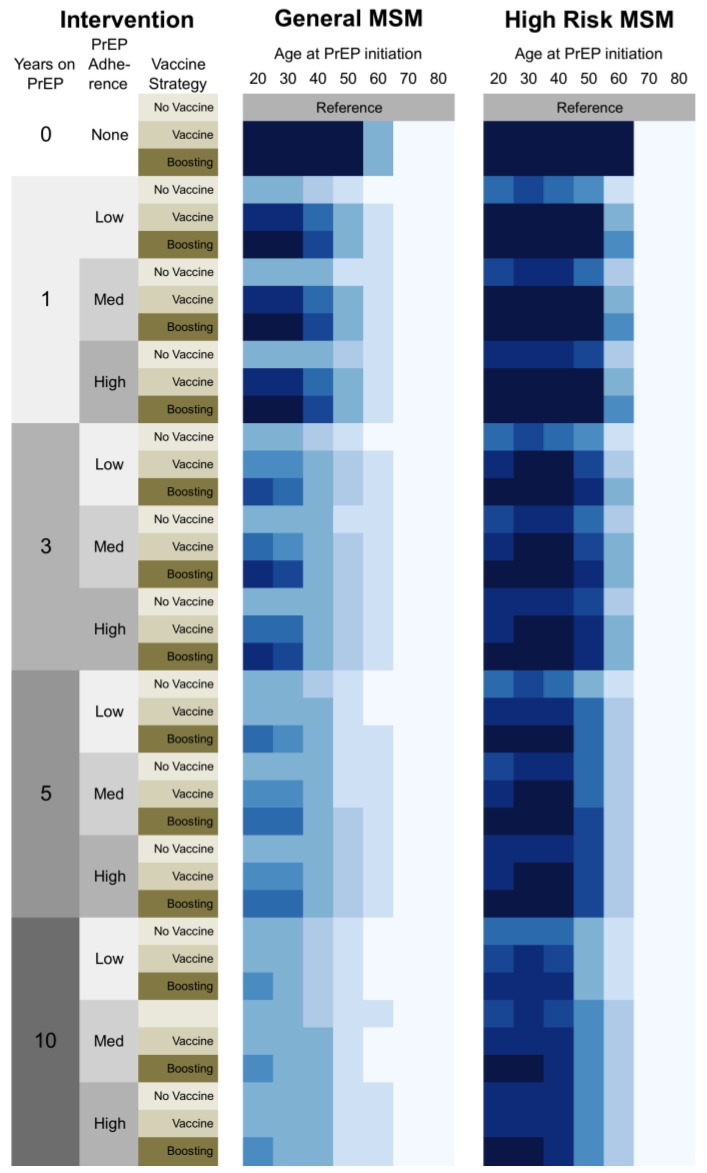
Sensitivity analyses of the cost-effectiveness of pairwise comparisons of scenarios versus standard care suggest that strategies would be more cost-effective with younger populations, higher-risk men, shorter duration on PrEP, and added HIV vaccine boosting. The darker blue color represents greater cost-effectiveness and the lighter color represents scenarios dominated or unlikely to be cost-effective as compared to the standard care.

Pairwise comparisons of policy-relevant scenarios for PrEP, HIV vaccines, and combination versus standard care are provided in a heatmap of cost-effectiveness (Figure 5). Vaccines only dominated PrEP if the HIV vaccine boosts restored a protective immunogenicity response at each injection. Vaccination without boosting gained 37% fewer QALYs and cost 30% more in lifetime HIV-related health care than re-boosting every 5 years. PrEP alone was cost-effective for high-risk men (with lifetime annual HIV incidence reaching a maximum of 8.9%) and had an ICER of $13,713 per QALY compared to standard prevention. With PrEP duration extended to 10 years, vaccination alone no longer dominated PrEP and it had an ICER of $776,786/QALY compared to the standard care. In this extended use scenario, the PrEP-vaccine combination dominated PrEP alone by extension. In high-risk men, 10 years of PrEP is projected to be cost-effective with an ICER of $64,159 per QALY vs. standard care. Figure 5 suggests that these HIV prevention interventions offer the greatest value in younger and higher-risk populations.

#### 3.2.2. Probabilistic Sensitivity Analysis

**Figure 6:**
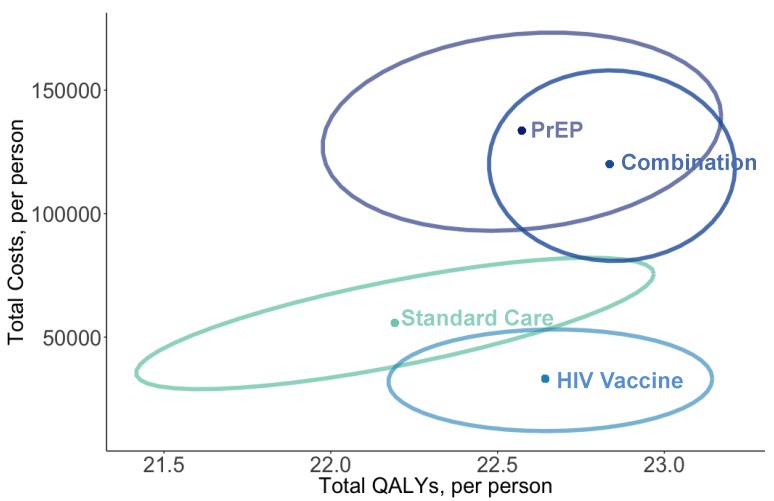
Distribution of incremental costs and QALYs per person among 1000 Monte Carlo simulations in the probabilistic sensitivity analysis. The ellipse represents 95% credible ranges around the average estimate for each strategy.

Consistent with the deterministic findings, HIV vaccines dominated standard care and PrEP alone in the probabilistic sensitivity analysis (Figure 6). PrEP alone cost $77,895 (95% credible range [CR] $42,095–$113,695) more per person than standard care and was the highest cost strategy. In comparison to HIV vaccines alone, adding PrEP for the combination strategy cost an additional $86,976 per person (95% CR $52,080–$121,853) and gained 0.19 QALYs (95% CR −0.06–0.44) per person on average. We estimated an average ICER of $696,318 per QALY (95% CR of −$584,780–$2 million) for the combination strategy versus HIV vaccines alone. The distribution of simulations in each strategy shows a shift in the distribution of simulations down (lower costs) and to the right (greater health) for the combination compared to PrEP alone.

## 4. Discussion

We projected the potential cost-effectiveness of HIV prevention strategies for MSM in the US after the future introduction of HIV vaccines. We found that HIV vaccination dominated PrEP alone (i.e., increased QALYs and reduced costs). A combination of PrEP with HIV vaccines provided the highest total QALYs but with substantial additional costs versus the other interventions: and it was unlikely to be cost-effective. However, the sensitivity analyses suggest that the combination strategy may be cost-effective for high-risk men provided the estimates for vaccine effectiveness from previous trials remain consistent in the ongoing pivotal trials. PrEP alone is not projected to be cost-effective in general MSM at the current PrEP prices.

PrEP costs too much to be cost-effective at the current prices. Potential options for PrEP to be cost-effective could include discounting the price by 50%, supported by findings of the threshold analysis, restricting the indication to higher-risk men, introduction of indication-specific pricing, or the entry of generic PrEP medications. Indication-specific pricing could accommodate one value-based charge and reimbursement for Truvada^®^ prescribed for HIV treatment and a second, lower, value-based price for Truvada^®^ prescribed for prevention [58]. If implemented, a larger population would be recommended for the cost-effective use of PrEP and HIV vaccines in combination. The anticipated reduction in PrEP costs with generic drug entry may be delayed if the recently approval drug tenofovir alafenamide fumarate (TAF, trade name Descovy^®^, Gilead Sciences Inc., Foster City, CA, USA) replaces TDF for PrEP. TAF may effectively extend the patent-life of Truvada^®^, capture new users, and help Gilead maintain its large market share of HIV drugs even after generic entry of TDF. Even if PrEP drugs were “free” for PrEP patients, the costs of implementation with quarterly clinic visits and STI diagnostic panels could substantially impact community clinic budgets. These results inform drug developers and suggest that long-acting injectable PrEP formulations could lower implementation costs with formulations capable of lasting greater than three months.

HIV vaccine success relies on either the durability of protection or the potential for boosting years later to elicit robust immunogenicity responses that correlate strongly with protection from infection. If PrEP drug costs are lowered, future HIV vaccine clinical trial designs may consider increasing sample sizes to evaluate the combined safety, efficacy, and potential synergy of HIV vaccines administered with PrEP. If HIV vaccines can more effectively reach disproportionately affected high-risk groups with little PrEP use, such as young Black and Latino MSM in the Southeastern United States [59], the availability of both products could substantially impact HIV incidence. Efficient implementation, defined as achieving the greatest health benefits under constrained healthcare resources, may be achieved through the recommendation of HIV vaccines for all MSM and PrEP for only some. The scenarios’ analysis informs vaccine research and development strategies with evidence that a moderately effective boosting dose could increase the potential market size for cost-effective administration by broadening the recommended target population from at-risk to the general population, and as a consequence increase potential revenue.

Our estimates for the cost-effectiveness of PrEP and HIV vaccines alone are consistent with results from other models including dynamic transmission models [21,60]. The PrEP-alone cost-effectiveness findings align with Juusola et al., who estimated PrEP for all MSM costs $216,480 per QALY gained (differing by 5% from our ICER for this population) [19]. Similarly, PrEP for injection drug users in the US was estimated by Bernard et al. to cost $253,000 per QALY gained [20]. For HIV vaccines alone, our cost-saving projections are consistent with Long et al. in the scenarios with similar assumptions [23]. The results from this analysis differ from a recent economic analysis of Canadian MSM where PrEP was cost-saving in almost all scenarios [61]. The different result may be due to lower Canadian drug costs and the selection of HIV incidence rates, as the Canadian study applied a constant number needed to treat (NTT) from a high-risk Peruvian population with 5% annual HIV incidence, while our analysis parameterized baseline infection rates to age-specific CDC HIV incidence in the USA. In the sensitivity analysis, if annual HIV incidence was increased to the same constant 5% rate, similar conclusions would be reached for the PrEP-alone cost-effectiveness. As HIV incidence is frequently a driver of the value of HIV prevention, the different sources of baseline transmission rates in each model may explain why different analyses have reached very different conclusions.

Our analysis had a number of limitations that warrant mention. First and foremost is the hypothetical nature of the efficacy estimates and attrition rates for long-term vaccine boosting and the combination of PrEP with vaccines. We considered a healthcare payer perspective and therefore did not include transmission dynamics to capture the indirect benefits of vaccination to others. As a consequence, our results are likely to underestimate the population-level health benefits from the prevention interventions. While the results of a dynamic population model can inform societal benefits over time, this study informs an individual patient or physician about the added costs and average gain in life expectancy and quality of life one 30-year-old man might gain over their lifetime if they choose to use PrEP and/or choose to be vaccinated. The model inputs are specific to the US and the results and conclusions are not transferrable to other settings. Considering the sensitivity of results to drug prices and HIV incidence, and how the values can differ greatly between countries, the cost-effectiveness of HIV vaccine and PrEP combinations should be analyzed for other settings using local inputs. The current incidence of HIV in MSM recommended to take PrEP is unknown, but we address this by scaling feasible ranges based on age trends in published data [32,62,63]. The model also assumes no behavioral disinhibition among intervention users, meaning an individual’s perception of protection from HIV will not lead them to increase risky choices. Future modeling studies should examine HIV vaccine uptake and the potential impact of the interaction with PrEP utilization as a complement or substitute.

## 5. Conclusions

Balancing the high cost and high effectiveness of PrEP with the potentially low cost and moderate effectiveness of HIV vaccines calls for the innovative design and testing of these products if combinations are planned for implementation. Achieving the ambitious milestones in the National Strategic Plan for the USA requires efficient spending of limited health care resources and research dollars. Early identification of high-value vaccine candidates and planning for optimal combinations with PrEP could extend many lives and reduce the burden of HIV in the USA.

## Figures and Tables

**Table 1:** Key model inputs.

Parameter	Value	Sensitivity Ranges		Reference
		Lower	Upper	
**HIV Incidence (Per 100 Person-Years)**				
25–34 year old MSM in United States	0.66%	0.56%	0.76%	[32,33]
35–44 year old MSM in United States	0.46%	0.38%	0.55%	[32,33]
45–54 year old MSM in United States	0.24%	0.19%	0.29%	[32,33]
High-risk scenario	2.0%	1.0%	4.0%	[50]
**Intervention Efficacy**				
Vaccine efficacy, 2 year average with 4 doses	31.2%	1.1%	52.1%	[14]
Decay parameter, *λ*_30_	−2.400	−2.037	−2.762	[44]
Vaccine Efficacy, 2 year average with 5 doses	50.0%	30.0%	70.0%	Assumed [51]
Decay parameter, *λ*_50_	−2.880	−2.400	−3.380	Calculated [44]
Vaccine boosting potential, ρ	100%	80%	100%	Assumed
PrEP Efficacy	86%	39.4%	98.5%	[52]
**Disease Progression**				
Probability of HIV symptoms, monthly	0.008	0.000	0.015	[30,53]
Probability of AIDS, monthly	0.081	0.009	0.700	[31]
Additional hazard of dying with HIV	1.770	1.170	2.550	[35]
AIDS mortality rate	0.43%	0.37%	0.51%	[36]
**Utilities**				
Healthy utility, age 30–39	0.918	0.912	0.925	[37]
Vaccine AE utility decrement	0.003	0.000	0.005	Assumed
PrEP AE utility decrement	0.008	0.000	0.020	Assumed
HIV Utility, CD4 >500	0.798	0.696	0.900	[30,38,40]
HIV Utility, CD4 200–500	0.780	0.767	0.793	[30,38,40]
AIDS Utility, CD4 <200	0.702	0.567	0.837	[30,38,40]
**Costs ^1^**				
Vaccine Price, per dose	$500	$100	$1000	Assumed
PrEP drug cost, 30-day supply	$1646	$893	$2000	[13,49]
PrEP visit cost, including lab tests	$208	$156	$260	[19]
HIV Care if CD4 >500, monthly	$1634	$1579	$1689	[48]
ART drug cost	$1211	$1172	$1251	[48]
Outpatient costs	$45	$43	$47	[48]
Other costs	$378	$364	$392	[48]
HIV Care, CD4 200–500, monthly	$1924	$1817	$2032	[48]
ART drug cost	$1158	$1103	$1212	[48]
Outpatient costs	$54	$51	$57	[48]
Other costs	$713	$663	$763	[48]
HIV Care, CD4 <200, monthly	$2558	$2334	$2783	[48]
ART drug cost	$1162	$1094	$1229	[48]
Outpatient costs	$62	$58	$67	[48]
Other costs	$1334	$1182	$1486	[48]

**^1^** Costs are presented in 2015 US dollars. Abbreviations: AE, adverse event; AWP, average wholesale price; ART, antiretroviral therapy; MSM, men who have sex with men; PrEP, preexposure prophylaxis.

**Table 2:** Base case outcomes per MSM receiving preventative care.

HIV Prevention Strategy	Total Costs ^1^	Total QALYs	HIV Infections	ICER ^2^ ($/QALY)
Standard Care	$51,926	22.057	170.7	Dominated
PrEP	$130,811	22.439	128.7	Dominated
HIV Vaccination	$30,870	22.580	88.3	Dominant
Combination: PrEP and Vaccine	$118,484	22.769	65.8	$463,448

^1^ Costs presented in 2015 US$ and discounted 3%; ^2^ ICERs present a ratio of incremental costs to incremental QALYs as compared to the next best option. Abbreviations: ICER, incremental cost-effectiveness ratio; PrEP, preexposure prophylaxis; QALYS, quality adjusted life-years.

## Abbreviations

The following abbreviations and corresponding units are used in this manuscript:

ART	Antiretroviral Therapy
AWP	Average Wholesale Price, $
CEA	Cost-Effectiveness Analysis
CPI	Consumer Price Index
CR	Credible Range
FTC	emtricitabine
ICER	Incremental Cost Effectiveness Ratio, $/QALY
LY	Life Years
MSM	Men Who Have Sex With Men
NNT	Number Needed To Treat
PrEP	Pre-exposure Prophylaxis
PSA	Probabilistic Sensitivity Analysis
QALY	Quality-Adjusted Life Year
TAF	tenofovir alafenamide fumarate
TDF	tenofovir disoproxil fumarate
USA	United States of America
VE	Vaccine Efficacy, %
WTP	Willingness to Pay, $

## Appendix A

**Table A1:** Description of HIV vaccine candidates in clinical trials.

Vaccine Component	RV144 Thai Trial			HVTN 702 South Africa Trial		
	DNA Prime	Protein Boost	Adjuvant	DNA Prime	Protein Boost	Adjuvant
Description	ALVAC-HIV recombinant canarypox vaccine, subtype B and E	AIDSVAX^®^ B/E bivalent HIV gp120 envelope glycoprotein vaccine, subtypes B and E	600 μg of alum adjuvant	Canarypox-based vaccine ALVAC-HIV subtype C	bivalent gp120 protein subunit vaccine, subtype C	MF59
Manufacturer	Developed by Virogenetics Corporation (Troy, NY) and manufactured by Sanofi Pasteur (Marcy-l'Étoille, France)	Originally manufactured by Genentech, Inc., further developed by VaxGen, Inc., later acquired by Global Solutions for Infectious Diseases (San Francisco, CA, USA)	VaxGen, Inc, no IP	Sanofi Pasteur	GlaxoSmithKline (GSK)	GSK
Trial Funding	Supported in part by an Interagency Agreement (Y1-AI-2642-12) between the U.S. Army Medical Research and Materiel Command and the National Institute of Allergy and Infectious Diseases and by a cooperative agreement (W81XWH-07-2-0067) between the Henry M. Jackson Foundation for the Advancement of Military Medicine and the U.S. Department of Defense. Sanofi Pasteur provided the ALVAC-HIV vaccine, and Global Solutions for Infectious Diseases (VaxGen) provided the reagents for the immunogenicity assays.			P5 members are National Institute of Allergy and Infectious Diseases (NIAID), the Bill & Melinda Gates Foundation (BMGF), the South African Medical Research Council (SAMRC), HIV Vaccine Trials Network (HVTN), Sanofi Pasteur, GSK, and the U.S. Military HIV Research Program. NIAID, BMGF, and SAMRC fund the P5. The National Institute of Allergy and Infectious Diseases (NIAID) is sponsoring and funding HVTN 702. Sanofi Pasteur and GSK are providing the investigational vaccines for the trial.		

**Table A2:** Impact inventory.

Sector	Type of Impact (Unit of Measure if Relevant)	Included in this Analysis from the Perspective of		
		Payer ☑	Health Care Sector	Societal
**Formal Health-care sector**	*Health Outcomes (Effects)*			
	Longevity effects (Life Years)	☑		
	Health-related quality of life (QALYs)	☑		
	Adverse events (QALYS)	☑		
	Secondary transmissions of infections			
	*Medical Costs*			
	Paid for by third-party payers ($)	☑		
	Paid for by patients out-of-pocket ($)			
	Future related medical costs to payers ($)	☑		
	Future related medical costs to patients ($)	NA		
	Future unrelated medical costs to payers ($)			
	Future unrelated medical costs to patients ($)	NA		
**Informal Health-care sector**	Patient time costs	NA		
	Unpaid caregiver time costs ($)	NA		
	Transportation costs ($)	NA		
**Productivity**	Labor market earnings lost ($)	NA	NA	
	Cost of unpaid lost productivity due to illness ($)	NA	NA	
	Cost of uncompensated household production ($)	NA	NA	
**Consumption**	Future consumption unrelated to health ($)	NA	NA	
**Social Services**	Cost of social services as part of intervention ($)	NA	NA	
**Education**	Impact of intervention on educational achievement of population	NA	NA	

Abbreviation: NA, not applicable; QALYs, quality-adjusted life years.

### Model Validation

We compared the mean total remaining life years for the cohort and compared this to the life expectancy of U.S. men. To support validation of the selected transition probabilities for HIV progression, we initialized HIV disease compartments with a cohort of newly infected patients and calculated the average remaining life-years. We subtracted this number from the average total life years for a scenario with no HIV infections and compared the difference to the estimated life years lost from HIV infection calculated by others. We validated the costs by comparing our estimated average cost of HIV care per infection to the lifetime cost of HIV calculated by Franham in 2013 and Schackman in 2015 [65,66].
